# Caribbean massive corals not recovering from repeated thermal stress events during 2005–2013

**DOI:** 10.1002/ece3.2706

**Published:** 2017-02-01

**Authors:** Benjamin Paul Neal, Adi Khen, Tali Treibitz, Oscar Beijbom, Grace O'Connor, Mary Alice Coffroth, Nancy Knowlton, David Kriegman, B. Greg Mitchell, David I. Kline

**Affiliations:** ^1^ The Bigelow Laboratory for Ocean SciencesEast BoothbayMEUSA; ^2^ The Scripps Institution for OceanographyUniversity of California San DiegoLa JollaCAUSA; ^3^ Marine Imaging LabLeon H. Charney School of Marine SciencesUniversity of HaifaHaifaIsrael; ^4^ Berkeley Vision and Learning CenterElectrical Engineering and Computer SciencesUniversity of California BerkeleyBerkeleyCAUSA; ^5^ Environmental Studies DepartmentColby CollegeWatervilleMEUSA; ^6^ Department of GeologyState University of New York at BuffaloBuffaloNYUSA; ^7^ National Museum of Natural HistorySmithsonian InstitutionWashingtonDCUSA; ^8^ Computer Science and EngineeringUniversity of California, San DiegoLa JollaCAUSA; ^9^ Smithsonian Tropical Research InstituteAnconRepublic of Panama

**Keywords:** climate change, coral bleaching, Coral reefs, *Orbicella franksi*, resilience and recovery, *Siderastrea siderea*, *Stephanocoenia michelini*

## Abstract

Massive coral bleaching events associated with high sea surface temperatures are forecast to become more frequent and severe in the future due to climate change. Monitoring colony recovery from bleaching disturbances over multiyear time frames is important for improving predictions of future coral community changes. However, there are currently few multiyear studies describing long‐term outcomes for coral colonies following acute bleaching events. We recorded colony pigmentation and size for bleached and unbleached groups of co‐located conspecifics of three major reef‐building scleractinian corals (*Orbicella franksi*,* Siderastrea siderea, and Stephanocoenia michelini*;* n* = 198 total) in Bocas del Toro, Panama, during the major 2005 bleaching event and then monitored pigmentation status and changes live tissue colony size for 8 years (2005–2013). Corals that were bleached in 2005 demonstrated markedly different response trajectories compared to unbleached colony groups, with extensive live tissue loss for bleached corals of all species following bleaching, with mean live tissue losses per colony 9 months postbleaching of 26.2% (±5.4 *SE*) for *O. franksi,* 35.7% (±4.7 *SE*) for *S. michelini*, and 11.2% (±3.9 *SE*) for *S. siderea*. Two species, *O. franksi* and *S. michelini*, later recovered to net positive growth, which continued until a second thermal stress event in 2010. Following this event, all species again lost tissue, with previously unbleached colony species groups experiencing greater declines than conspecific sample groups, which were previously bleached, indicating a possible positive acclimative response. However, despite this beneficial effect for previously bleached corals, all groups experienced substantial net tissue loss between 2005 and 2013, indicating that many important Caribbean reef‐building corals will likely suffer continued tissue loss and may be unable to maintain current benthic coverage when faced with future thermal stress forecast for the region, even with potential benefits from bleaching‐related acclimation.

## 1 Introduction

Mass coral bleaching episodes, characterized by the loss of photosynthetic endosymbiotic algae (Genus *Symbiodinium*), are caused by sustained elevated water temperature events and are becoming more frequent and severe with global warming (Hoegh‐Guldberg, 1999; Mcwilliams, Cote, Gill, Sutherland, & Watkinson, 2005; Pandolfi, Connolly, Marshall, & Cohen, 2011; Walther et al., 2002). There has been a large body of research describing the biological effects of coral bleaching, including reduced short‐term growth (Baird & Marshall, 2002; Cantin, Cohen, Karnauskas, Tarrant, & Mccorkle, 2010; Goreau & Macfarlane, 2000), reduced reproduction (Baker, Glynn, & Riegl, 2008; Szmant & Gassman, 1990; Ward, Harrison, & Hoegh‐Guldberg, 2002), increased mortality (Jokiel & Coles, 1977; Miller, Waara, Muller, & Rogers, 2006), increased disease outbreaks (Altizer, Ostfeld, Johnson, Kutz, & Harvell, 2013; Kline, Kuntz, Breitbart, Knowlton, & Rohwer, 2006; Vega Thurber et al., 2008), and colony fragmentation (Elahi & Edmunds, 2007; Meesters et al., 1996; Mumby, 2016). However, there are few multiyear demographic studies. Multiyear studies are needed to determine postdisturbance colony trajectories over a sufficient period to determine persistent effects (both positive and negative) associated with an acute bleaching event. These studies are necessary for improving predictions of how bleached scleractinian communities will respond on annual and decadal scales (Logan, Dunne, Eakin, & Donner, 2014) and how a warmer future may drive long‐term changes affecting coral community composition and ecosystem structure.

The long‐term effects of bleaching events on coral colony growth and coral reef community structure are still not clear (Baker et al., 2008; Pandolfi et al., 2011). Bleached colonies can suffer relatively immediate bleaching‐related mortality (Brandt, 2009; Carilli, Norris, Black, Walsh, & Mcfield, 2009), but also can potentially develop enhanced resilience as a result of acclimation to thermal stress (Buddemeier & Fautin, 1993). The adaptive bleaching hypothesis (ABH) suggests that changes in the coral photosymbiont populations following a bleaching event may allow some corals to re‐establish a symbiosis with different strains of endosymbiotic dinoflagellates, resulting in a holobiont better suited to the altered environmental conditions (Brown, Dunne, Phongsuwan, Patchim, & Hawkridge, 2014). This hypothesis does not explicitly state that this will result in enhanced growth or improved survival rates, but opens the possibility of such, as well as the possibility of enhanced resistance to bleaching when faced with repeated thermal stress.

Recent laboratory studies indicate that repopulation of the coral endosymbionts with a different symbiont community following a bleaching and recovery response is necessary to increase heat tolerance for individual colonies (Silverstein, Cunning, & Baker, 2015). In this study, coral colonies that previously bleached were less affected by subsequent thermal stress. In contrast, the same adaptive response was not seen from past exposure or acclimation to warmer temperatures prior to subsequent thermal stress sufficient to potentially cause bleaching. Given that individual coral colonies have the potential for highly stochastic responses (Baird, Bhagooli, Ralph, & Takahashi, 2009; Mydlarz, Mcginty, & Harvell, 1999), even for conspecifics exposed to the same environmental stimuli, the identification of these differences in response, recovery, and future resistance related to bleaching is necessary for estimates of larger population or community aggregate responses.

**Figure 1:**
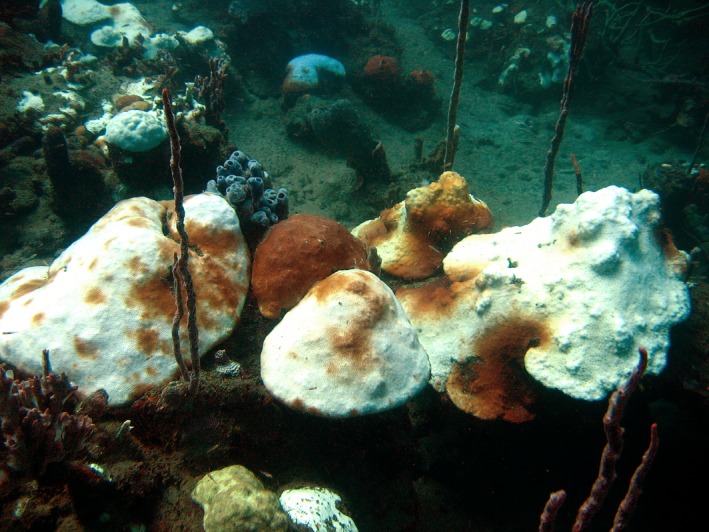
Bleaching in 2005 in Bocas del Toro, Panama. This image shows typical bleaching in October 2005 on the fringing reef inside Bahia Almirante, Bocas del Toro. A number of species are seen in this landscape, and also both the manner in which conspecifics (in this image, *Orbacella franksi*) can exhibit very different individual colony responses to identical thermal stress, as well as how partial bleaching can be seen over single colonies. Both of these phenomena were significant for our analysis

Our research investigates the different long‐term responses of individual coral colonies stemming from thermal stress exposure alone vs. those following expressions of acute visible bleaching, and differs from Silverstein et al. (2016) in that it extends that time frame for response observation to 89 months, and importantly provides validation of this phenomenon in a field setting following a natural bleaching event (Figure 1). These three species are all are massive, hermatypic, relatively slow‐growing corals, and all have similar physiological energetics (i.e., zooxanthellate) and are broadcast reproduction (Szmant, 1986), leading to relatively high genetic connectivity across the Caribbean basin (Nunes, Norris, & Knowlton, 2015). Skeletal extension rates are also roughly similar, with range of 1.4–10.0 mm/year for *O. franksi* (Huston, 1985), 1.8–3.8 mm/year for *S. michelini* (Hubbard & Scaturo, 1985), and 2.7–9.3 mm/year for *S. siderea* (Huston, 1985).

We sought to investigate the long‐term massive coral species colony‐level responses to the 2005 Caribbean mass bleaching disturbance over a sufficiently long period to demonstrate both initial and delayed mortality from bleaching and/or thermal stress, to allow for recovery of colony function to stable predisturbance rates, and to potentially expose differences in outcome stemming from organismal or symbiotic adaptive responses. We focused on massive type, hermatypic (or reef‐building) species, as these are the key ecosystem architects in these systems, creating essential habitat for the reef community. An expanded understanding of the long‐term response of corals to specific disturbances, in this case bleaching, is essential for guiding how we structure management and conservation goals. Our quantification of recovery responses is also central for gauging expectations for recovery and persistence of coral reef ecosystems, which is particularly relevant as the frequency and intensity of some disturbances, including elevated sea surface temperature, are currently changing in response to global climate change (Hoegh‐Guldberg & Bruno, 2010), and massive coral bleaching events may soon become much more frequent or effectively continuous (Pandolfi et al., 2011).

## 2 Materials and Methods

Making extended repeat *in situ* time series measurements of coral condition over time for massive‐type scleractinian colonies presents several practical and logistical challenges. First, coral colonies have relatively slow growth rates (1–10 cm/year) and require long observation times to record detectable changes (Lough & Cantin, 2014), and second the predominant method for determining growth commonly utilizes potentially destructive methods such as radiographic sclerochronology (DeLong et al., 2013). Radiographic sclerochronology determines coral colony skeletal extension rates by either coring or slicing the coral skeleton, and measuring growth bands either visually or with X‐ray methods, utilizing either simple planar or computed tomographic analysis. These techniques can be at a minimum partially destructive, but nonlethal, to larger live colonies but can be wholly destructive of smaller colonies. As our goal was to measure postdisturbance mortality/growth for an extended number of years repeated coring was simply not practical on an annual basis for the small to medium‐sized colonies in our sample group, due to the potentially deleterious effect on the coral subjects. Furthermore, we hoped to relate colony growth with observed recovery and status of the surface tissue condition for each of those years, and skeletal analysis does not provide information on surface tissue condition. We therefore recorded planar area of live tissue and extent of bleaching and partial bleaching within that live tissue area through the noninvasive analysis of underwater photographs, taken approximately annually.

### 2.1 Study site

**Figure 2:**
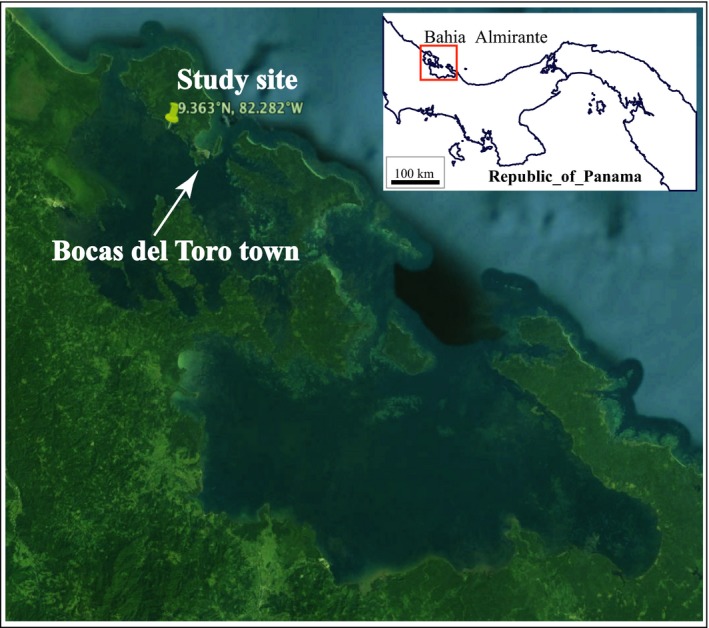
Study site in Caribbean western Panama. The study reef was located within the protected Bahia Almirante embayment, an area of extensive fringing reef development. Our site was about 3 km from the nearest densely settled municipal area (Bocas del Toro)

This study was conducted on a protected fringing reef near Punta Caracol on the western (leeward) side of Isla Colon, Bocas del Toro, Panama (9.363°N, 82.282°W; Figure 2). The reef is located within the Bahia Almirante embayment, an area of extensive but patchy coral cover, with coral development along a slope from the surface to 20 m (Guzman, PaG, Lovelock, & Feller, 2001). Coral species diversity and cover are high for Panamanian reefs, and are typical for well‐developed coral reefs in the western Caribbean (Guzman & Guevara, 2005). This site was chosen to represent as much as possible an unaffected inshore reef site, to assess as nearly as possible the impact primarily from the thermal disturbances. However, the embayment does have high environmental variability, as it is heavily influenced by both oceanic water input as well as high freshwater input (Collin, Huber, Macintyre, Ruetzler, & Ruiz, 2009), and water clarity is lower than many reef sites due to higher nutrient and chlorophyll concentrations, contributing to shallower reef development (Kaufmann & Thompson, 2005). Furthermore, coastal development and anthropogenic eutrophication are not insignificant in the town of Bocas del Toro, but our study site is located ~3 km to the northwest of the town, with reduced influence in this location from anthropogenic development/disturbance. All field work was conducted under the aegis of the Smithsonian Tropical Research Institute, with scientific permits for working with protected species were obtained from the Direccion de Areas Protegidas de Vida y Silvestre, Autoridad Nacional de Ambiente, Republica de Panama, in Balboa, Ancon, Panama.

### 2.2 Thermal stress determination

In 2005, the Caribbean basin was subjected to the highest water temperatures recorded to date for this area (Eakin et al., 2010), resulting in extensive bleaching across the basin (Lajeunesse, Smith, Finney, & Oxenford, 2009; Miller et al., 2006; Whelan, Miller, Sanchez, & Patterson, 2007). Thermal stress conditions of lower magnitude were also experienced in 2010 (Guest et al., 2012), as was widespread bleaching. A much more detailed analysis of this methodology and the calculations of thermal stress conditions for Bocas del Toro for both the 2005 and 2010 bleaching events can be found in Neal et al. (2010); for the purpose of this manuscript, we updated this previous record through late 2014, but did not alter the methodology or algorithms.

Local temperature conditions and estimations of coral thermal stress in the study area were assessed using an ongoing local in situ depth‐stratified sea temperature time series recorded at three depths (4, 10, and 20 m) using calibrated HOBO StowAway TidbiT and HOBO Water Temperature Pro V2 instruments (Onset Computer Corp., Bourne, MA, USA), collected as a part of the Smithsonian Tropical Research Institute (STRI) Physical Monitoring Program. Data processing followed methods developed by the National Oceanic and Atmospheric (NOAA) Coral Reef Watch (CRW) program, which utilizes remotely sensed nighttime sea surface temperatures (SST), producing as primary outputs the calculated coral stress metrics Coral HotSpot and Degree Heating Weeks (DHW; expressed in units of °C weeks). Thermal stress conditions are defined as HotSpot values 2.0°C or greater, and the onset of potentially damaging coral bleaching was defined to begin at DHW values of 4°C weeks or greater.

### 2.3 Measurements of bleaching response and recovery

Conspecific groups of bleached and unbleached individual colonies of the three target species (*Orbicella franksi, Siderastrea siderea,* and *Stephanocoenia michelini; n* = 198 total*)* were selected, tagged, and monitored mortality and growth outcomes on approximate annual intervals for nearly 8 years after the major 2005 thermal stress event. This time span of observations also fortuitously included a second major thermal stress event in late 2010, allowing for evaluation of response of previously exposed colonies with known bleaching history to repeat bleaching events. Metrics of colony response aimed to elucidate: (1) species‐specific rates of partial or total colony mortality following the 2005 thermal anomaly; (2) recovery time for colonies to return to steady state or positive growth of tissue area; (3) differences between species and between bleached and unbleached conspecific groups in postdisturbance tissue growth rates; and (4) tissue loss following the second 2010 thermal stress event. All of these metrics have implication for predicting decadal‐scale changes in coral reef community composition.

The three coral species chosen for this study are all are massive type, hermatypic, relatively slow‐growing corals, and all have similar physiological energetics (i.e., zooxanthellate) and are broadcast reproduction (Szmant, 2014), leading to relatively high genetic connectivity across the Caribbean basin (Nunes et al., 2015). Skeletal extension rates are also roughly similar, with ranges of 1.4–10.0 mm/year for *O. franksi* (Huston, 1985), 1.8–3.8 mm/year for *S. michelini* (Hubbard & Scaturo, 1985), and 2.7–9.3 mm/year for *S. siderea* (Huston, 1985). They were chosen to be characteristic of structure‐building species contributing to both the creation of living habitat as well as long‐term carbonate reef accretion.

Photographic monitoring was begun in early October 2005 during the mass bleaching event. Colonies were selected opportunistically in the same general area along three depth transects (<4, 7–10, and 10–13 m) while on SCUBA. Colony selection was not entirely random, due to limited numbers of suitable subjects along the depths transects, but effort was made to find a comparably similar number of representative samples of bleached and unbleached colonies for the three species in each transect, and it should be noted that the *S. michelini* population *in situ* was predominantly bleached, and the *S. siderea* population *in situ* was predominantly unbleached, requiring somewhat wider examination of the area to obtain representative populations for the two bleaching status groups. Effort was made to find proximally located pairs (bleached and unbleached colonies), to minimize the impact of different flow patterns, source water, and other localized environmental variables, with the entire sample located along an approximately 150‐m transect on the same reef section. Selected colonies were permanently tagged with white PVC plastic tags with stamped numbers, fastened to dead areas of the substrate with stainless steel nails. These tags were removed and replaced three times with flexible plastic, laser engraved, numbered cattle tags (Allflex, Dallas, TX, USA) over the course of the time series to ensure that the numbers were legible and that tags were not broken or lost. Colonies were photographed immediately after selection and tagging, as the initial time point for the time series. These photographs were not all taken on the same day, due to the practicality of locating, tagging, and imaging numerous colonies, but were all marked and sampled during the acute bleaching period over approximately 2 weeks, near the peak of water temperatures during the 2005 thermal stress event, and are thus considered a single time point for analysis.

All colonies were revisited for additional photography at 6, 9, 22, 34, 46, 58, 71, and 89 months after the initial observations in October 2005. Images were taken over the period with a variety of consumer‐grade underwater cameras, with later years using a Canon 5D MkII 23 megapixel DSLR, with a Canon EF 17–40 mm lens with dome port with extender fitted to minimize radial distortion (Treibitz, Schechner, Kunz, & Singh, 2012). Underwater lighting was provided as needed with a variety of equipment, in later years using dual Sea&Sea YS250 strobes, and images were color‐corrected for spectral water column effects.

**Figure 3:**
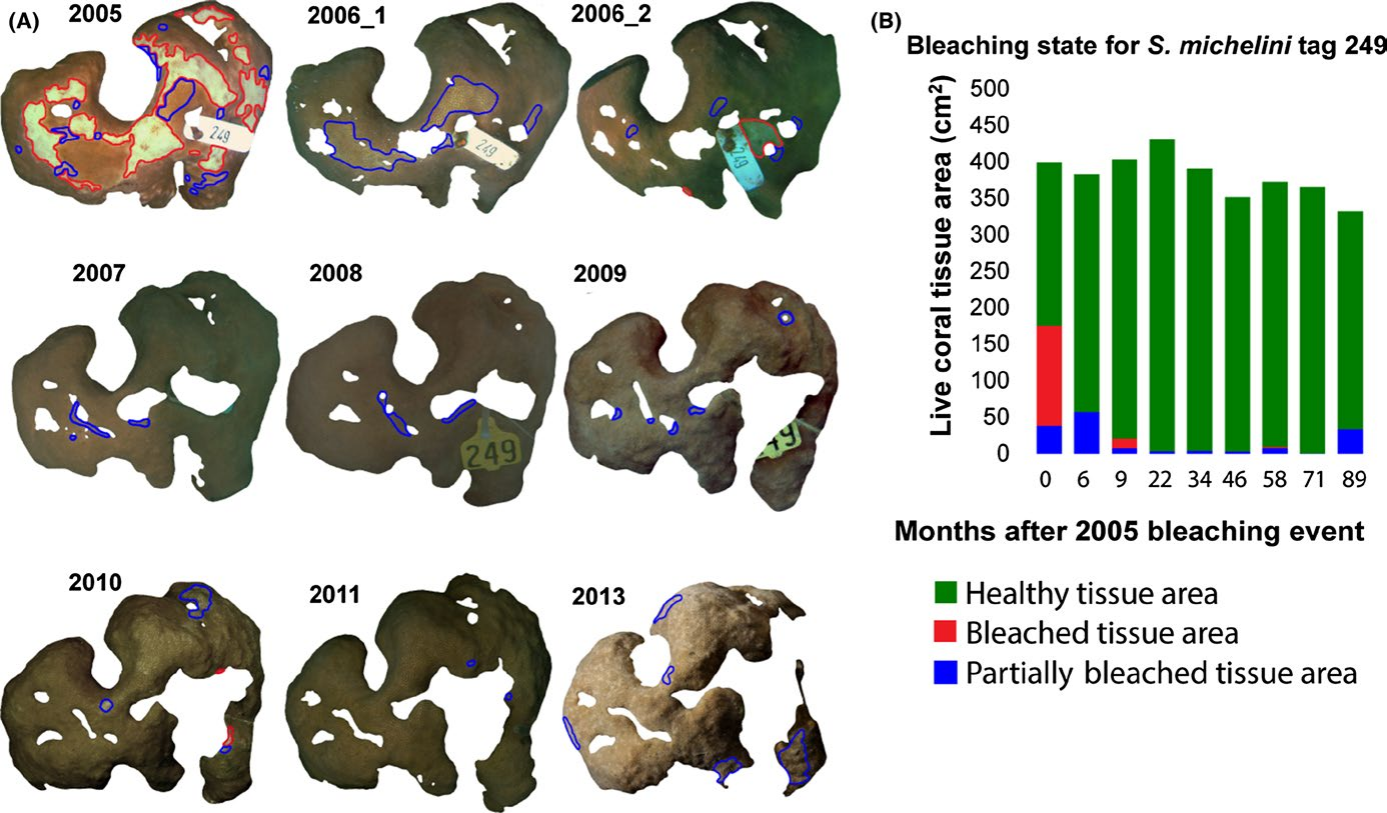
Segmented live coral tissue areas for a single example colony from 2005 to 2013. (A) Healthy pigmented portions of live tissue area are screened from the color‐corrected background images and outlined in green, with partially bleached tissue in blue and bleached areas in red. Some interannual differences in image color are due to water conditions, lighting, or camera sensitivities. (B) Bar plot shows changes in bleaching state across the time frame of the study for this colony. This example colony (Tag #249) did not demonstrate massive tissue loss, recovering quickly from significant bleaching in 2005, but did experience long‐term patchy tissue mortality, largely localized to previously bleached areas

Colony photographs were analyzed for projected planar area (in units of cm^2^) and pigmentation condition of live coral tissue, expressed as a percent of the total live area (with any patches of dead exposed skeleton surrounded by live tissue removed from the live area analysis), following Neal et al. (2013). All photographs included a color and size reference in the image and were analyzed with a custom MATLAB‐based image segmentation tool (http://vision.ucsd.edu/content/coral-colony-segmentation-and-area-measurement-tools), resulting in planar area measurements of live coral tissue, dead area on the colony, and bleached and partially bleached tissue (as subsets of the live area measurement) (Neal et al., 2013). Bleached tissue was defined as tissue exhibiting nearly completely white areas, and partially bleached tissue as that with residual pigmentation remaining in the bleached areas (also referred to as paling). These two designations could be, and often were, present on the same colony in complex patterns (Figure 3).

### 2.4 Colony sample sets

Two coral colony size classes were defined postanalysis for each species (large and small), with the class division based on the mean size for each species in the sample set. A total of 52 *O. franksi* colonies were tagged and photographed in 2005, with 37 visibly bleached, and 15 unbleached. Twelve colonies were at a water depth of 4 m or less, 16 at 7–9 m depth, and 24 at 10–12 m. Colonies ranged in planar area from the smallest at 108 cm^2^ to the largest at over 3,400 cm^2^, with a mean size of 457.5 cm^2^ (±63.4 *SE*). Thirty‐two colonies were classified as large, and 20 as small. In the case of this species only, some large individual colonies (over 1,500 cm^2^) were spatially sub‐sampled for bleaching state, as measurement of these large colonies in their entirety was not practical with these photographic methods; bleaching extent and growth measurements for these colonies were all included in the large colony group dataset.

Sixty‐eight *S. michelini* colonies were tagged and photographed in 2005, with 62 exhibiting visible bleaching, and six unbleached. Thirteen colonies were in 4 m water depth or less, 24 in 7–10 m depth, and 31 in 10 m. Colonies ranged in planar area from 48 to 403 cm^2^, with a mean size of 239.9 cm^2^ (±18.8 *SE*). Twenty‐five colonies were classified as large and 43 as small.

Fifty‐eight *S. siderea* colonies were tagged and photographed in 2005, 25 exhibiting visible bleaching in 2005, and 33 unbleached. Twenty‐one colonies were in 4 m of water or less, 21 in 7–10 m depth, and 16 in 10–13 m. Colonies ranged in planar area from 39 to 1133 cm^2^, with a mean size of 383.9 cm^2^ (±37.5 *SE*). Twenty‐four *S. siderea* colonies were classified as large and 34 as small.

### 2.5 Comparisons of bleaching severity, mortality and recovery

Analysis and discussion of the time series is divided into four time points/sections, defined as: (1) initial 2005 bleaching extent; (2) the postbleaching response period; (3) the bleaching recovery period; and (4) the 2010 bleaching event response period. Details of these divisions are given in Table 2.

The initial bleaching period is when bleaching was acutely and visibly manifested; the response period is when direct bleaching effects may no longer be visible but are manifested in disturbance‐associated tissue loss (whole or partial mortality), whole‐colony mortality, and pigment changes. Delineation of the response period was determined post hoc from observed recovery trajectories in this study (i.e., when tissue began to increase in size again following the losses from the disturbance event, and largely recovered visible pigmentation), along with estimations from the literature, including observations of post bleaching zooxanthellate densities recovering within 12 months (Fitt, Spero, Halas, White, & Porter, 1993), significant reduction of visual Bleaching Index for many taxa of massive corals within 9 months (Mcclanahan, 2004), and near complete recovery of pigmentation in Caribbean *Montastrea ssp*., (now genus *Orbacella)* within 6 months (Goreau, Mcclanahan, Hayes, & Strong, 1990). The recovery period was thus defined in this study as the first 9 months following the disturbance, and trajectories calculated for this period include three sets of observations and size data, the first being the initial set of images, and two taken 6 and 9 months later. It must be recognized that recovery is not a uniform process, nor is the definition of a “recovered coral”, so this temporal delineation for this analysis must be taken as a heuristic definition and an estimate for examining groups of colonies, and not as an absolute period for all individual colonies to exhibit recovery from this disturbance. The recovery period was defined as the time when thermal conditions maintained near the long‐term mean, with no notable sea surface temperature variations or other known notable environmental events that may have affected the subject corals. This period lasted 4 years and includes four observations, taken 22, 34, 46, and 58 months after the initial disturbance. The 2010 bleaching response period was defined as the two final photographic observations taken 71 and 89 months after the initial 2005 bleaching event. These final two observations, beginning some 11 months after a second thermal stress event in late 2010, represent the response to this second elevated temperature event, but not the acute short‐term response. This event took place approximately 60 months after the 2005 event and was of lesser disturbance magnitude than the 2005 event, but still severe enough to cause widespread bleaching warnings and coral bleaching (Guest et al., 2012). The peak of this disturbance event took place approximately 4–6 weeks after our 2010 imaging trip. There is consequently (and unfortunately) no direct maximum acute bleaching response record for this second event, as we had with the first, but there are before and after photographs for the event, and remotely and locally sensed records of the magnitude of thermal stress. Given the primary desire to observe and quantify long‐term colony mortality, the lack of direct observation of maximal bleaching is not a critical issue, but does assume that mortality seen following the thermal stress event is associated with stress (and consequent bleaching). We have made this assumption, but given the multitude of environmental stressors affecting coastal coral reef systems, assigning attribution of effect to a given single environmental cause must be done cautiously.

### 2.6 Statistical analyses

For comparisons of initial bleaching reaction in 2005, within‐species differences in both bleaching and partial bleaching extent between the large and small size groups were evaluated with Student's *t* tests (*α* = .05). As there was an *a priori* division in the selection of bleached and unbleached individuals, no statistical evaluation was made of differences between bleached and partially bleached areas for these groupings, although these means are reported as confirmation that the three unbleached sample sets had little visible effect from the thermal stress, and thus do in fact represent a distinct group of colonies in terms of visible physiological response to thermal stress.

Annual change rates for live tissue area for both response and recovery periods are calculated in two ways: First, an annualized % change calculated from linear monthly regression coefficients across multiple observations, and second as annualized % mean change in individual colony planar size calculated from difference between beginning and ending observations for the time periods. There was close agreement between these two methods, and we primarily use the results from the regression analysis for discussion. Recovery time for colonies to return to positive tissue growth was determined by fitting a second‐order polynomial regression and calculating the time when slope = 0, and periods for recovery to initial live areas were determined through basic compound interest calculations.

## 3 Results

### 3.1 Thermal stress conditions

**Figure 4:**
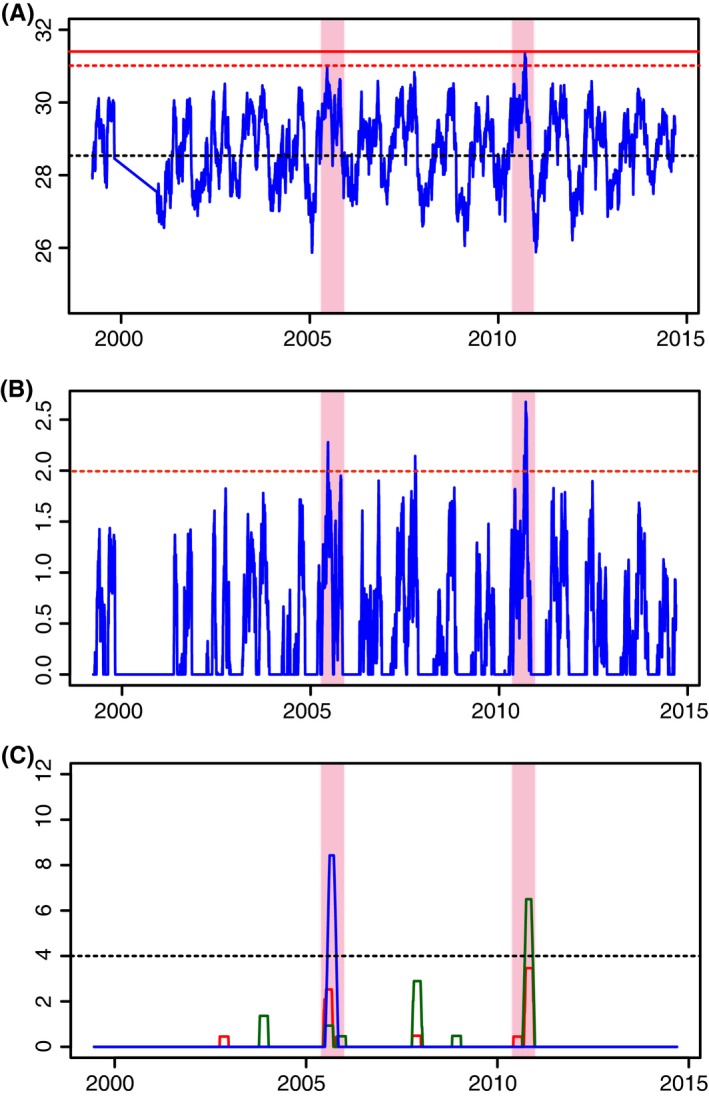
Sea temperatures and indices of thermal stress in the Bocas del Toro region from 1999 to 2014. (A) Daily mean temperatures for 4 m depth, with the black dashed line indicating the long‐term mean of 28.68°C, the red dashed line indicating the nominal bleaching threshold of 31°C, and the solid red line indicating the highest mean daily temperature recorded of 31.61°C. (B) HotSpot metric for 1999–2005, with the dashed red line indicating bleaching threshold of 2.0. (C) Cumulative bleaching stress for three depths, with blue indicating 20 m, red 10 m, and green 4 m depths. Black dashed line indicates 4°C weeks, the bleaching risk threshold defined by NOAA CRW. Light red shading indicates the two primary bleaching periods in 2005 and 2010 on all three plots

High levels of thermal stress in the period 2005–2014 occurred primarily in 2005 and 2010 (Figure 4). The highest absolute near‐surface (4 m depth) daily mean temperatures on the reef (inside Bahia Almirante) were seen in 2010, with a maximum of 31.61°C. In 2005 and 2010, individual water temperatures greater than or equal to 31°C were recorded, with near this level seen in 2007. This threshold is often considered a threshold for risk of bleaching, but this definition is locally dependent, with the same species of corals in different areas demonstrating acclimation to local conditions (Manzello, Berkelmans, & Hendee, 2007). Total calculated stress for these 2 years was highest as well (Figure 4), with the HotSpot metric exceeding the 2.0 threshold in 2005, 2007, and 2010, and the DHW metric exceeding the 4°C weeks threshold only in 2005 and 2010. The long‐term mean for this area at 4‐m depth was 28.68°C; from 2005 to 2015, the mean was 28.72°C, while for the earlier 1999–2005 the mean was 28.60°C. Minimum daily mean recorded for 4 m depth for the period 1999–2014 was 25.56°C.

### 3.2 Initial bleaching response in 2005 by species:

*Orbicella franksi* colonies demonstrated no whole‐colony bleaching, as this species generally exhibited patchy bleaching, with individual colonies showing a complex patterned mix of affected and normally pigmented tissue (Table 2). The bleached group had a mean bleaching extent per colony of 14.4% (±2.4 *SE*) of live tissue and a mean partial bleaching extent of 18.8% (±1.8 *SE*). The unbleached group had a mean bleaching extent of 0.7% (±0.2 *SE*) and a mean partial bleaching extent of 1.6% (±0.6 *SE*). These small amounts of bleached tissue in the unbleached group were commonly from small spots of bleached and partially bleached tissue, usually on growth edges. The single most affected colony was 67.9% bleached, and several colonies from the unbleached group were <1% bleached. Colonies in the large colony size group (*n* = 32) had a mean bleached area of 11.5% (±2.5 *SE*) of live tissue, and small colonies (*n* = 20) had a mean bleached area of 8.9% (±2.0 *SE*), a nonsignificant difference (*t *= 0.058, *df *= 50, *p *=* *.56) (Table 1). Partially bleached areas were also not significantly different (*t *=* *0.76, df = 50, *p *=* *.45) between the two size groups; large colonies had a mean of 15.1% (±2.7 *SE*), and small colonies 12.4% (±2.8 *SE*).

**Table 1:** Analysis time periods

Time periods	Time covered	Sampling times (months elapsed since 2005 bleaching)	# of image sets taken in this period	Notes
Initial 2005 bleaching extent	October 2005	0	1	Acute coral bleaching observed, highest water temperatures recorded. All images taken within a 10‐day period
2005 Postbleaching response period	October 2005–July 2006	6, 9	2	Bleaching still visible, but reducing. Mortality highest in this period
Recovery period	July 2006–August 2010	22, 34, 46, 58	4	Thermal conditions stable, low coral mortality. Second bleaching event happened at month 60 (October 2010)
2010 Postbleaching response period	September 2011–March 2013	71, 89	2	Local bleaching reported following month 60, first postbleaching images taken at month 71

Analysis and discussion of the study time frame was divided into these periods, relative to both coral state and acute thermal stress periods. The recovery period was selected post hoc for this analysis and may vary for different regions and species.

*Stephanocoenia michelini* exhibited extensive whole‐colony bleaching, and some individual colonies show a mix of bleached and normally pigmented tissue (Table 2). The bleached group had a mean bleaching extent per colony of 38.3% (±4.9 *SE*) of live tissue, and a mean partial bleaching extent of 19.4% (±2.7 *SE*) (Figure 1). The unbleached group had a mean bleaching extent of 0.1% (±0.05 *SE*) and a mean partial bleaching extent of 5.2% (±1.4 *SE*). Completely unaffected colonies (with no bleaching or partial bleaching) of *S. micheleni* were difficult to locate in the 2005 bleaching event. Several colonies were 100% affected (either bleached or partially bleached), and turf algal growth was visible on some colonies in these initial images, indicating that these colonies may have bleached earlier in the bleaching season in the event and were already experiencing mortality over parts of their surface. Colonies in the large colony size group (*n* = 25) had a mean bleached area of 35.9% (±6.1 *SE*) of live tissue, and small colonies (*n* = 43) had a bleached area of 39.2% (±5.9 *SE*), a nonsignificant difference (*t *=* *0.70, *df *= 66, *p *=* *.49) (Table 1). Partially bleached areas were also not significantly different between the two size groups (*t *=* *0.56, *df *= 66, *p *=* *.59); large colonies had a mean of 20.6% (±2.7 *SE*), and small colonies 12.4% (±2.8 *SE*).

**Table 2:** Stress responses by size class in 2005

Species	Size class	*n*	%BL in 2005	BL *p* value: large versus small (*α* = 0.05)	%PB in 2005	PB *p* value: large versus small” (*α* = 0.05)
*Orbicella franksi*	Large	32	11.5 ± 2.5	.56	15.1 ± 2.7	.45
	Small	20	8.9 ± 2.0		12.4 ± 2.8	
*Stephanocoenia michelini*	Large	25	35.9 ± 6.1	.49	20.6 ± 2.7	.59
	Small	43	39.2 ± 5.9		12.4 ± 2.8	
***Siderastrea siderea***	**Large**	**24**	**1.6 ± 0.32**	**<.001**	**2.6 ± 0.5**	**.0005**
	**Small**	**34**	**11.2 ± 1.9**		**10.3 ± 1.9**	

Bleaching (BL) and partial bleaching (PB) extent for large and small colonies of each species in 2005. Large colonies were defined as those greater than the mean size for each species and small as those less than the mean. Only ***S. siderea*** showed significant differences between the two size classes (in bold), with larger colonies less likely to bleach or partially bleach after a thermal stress event.

*Siderastrea siderea* was generally much less severely bleached, and no colonies exhibited whole‐colony bleaching (Table 2). Colorations changes were more gradual and subtle than for the other two species, generally occurring in larger patches over the surface of the colonies. The bleached group had a mean bleaching extent per colony of 13.1% (±4.6 *SE*) of live tissue, and a mean partial bleaching extent of 12.8% (±3.1 *SE*). The unbleached group had a mean bleaching extent of 0.2% (±0.04 *SE*) and a mean partial bleaching extent of 0.9% (±0.4 *SE*). Unlike the other two species, *S. siderea* showed a significant difference in bleached tissue extent between the two size classes of the bleached group (*t *=* *5.7989, *df *= 56, *p *<* *.001); colonies in the large group (*n* = 24) had a mean bleached area of only 1.6% (±0.32 *SE*) of live tissue, with small colonies (*n* = 34) bleached 11.2% (±1.9 *SE*) (Table 1). Partially bleached areas were also significantly different between the two size groups (*t *=* *3.6830, *df *= 56, *p *=* *.0005), with large colonies having a mean of 2.6% (±0.5 *SE*), and small colonies 10.3% (±1.9 *SE*). It should be noted that for *S. siderea* our estimate of bleaching extent also explicitly applied only to the selected individuals in the visibly bleached group, and the extent of bleaching across the entire population would be considerably less than the amount reported here as we preferentially searched for affected colonies to sample both bleached and unbleached colonies, and thus our bleaching rates possibly overestimate the extent of bleaching in the natural population.

### 3.3 Tissue loss and mortality following bleaching

The *O. franksi* bleached group (*n* = 37) lost an average of 26.2% (±5.4 *SE*) of live tissue area per colony in the first 9 months following the bleaching event (Table 2). Greatest tissue loss for this group was recorded 34 months after the disturbance, with mean colony live tissue mortality of 35.0% (±7.8 *SE*) compared to their 2005 extent. The unbleached group did not lose tissue over any interval in this period, gaining 2.8% (*SE* ±5.6) in live area by month 58. Three *O. franksi* colonies died completely in the first nine months (8.1% of the bleached colony sample group) following the 2005 thermal stress event, all of which were bleached in 2005, with an average bleaching area for the total mortality group of 18.5% (±5.2 *SE*), not significantly different than the mean for the bleached group itself (*t *=* *0.4757, *df *= 38, *p *=* *.6370), indicating that bleaching extent alone is not a strong predictor of total colony mortality. For comparison, two additional colonies died over the next 49 months.

Bleached *S. michelini* colonies (*n* = 62) lost an average of 35.7% (±4.7 *SE*) of live tissue area per colony in the first 9 months following the bleaching event (Table 2). Tissue loss peaked 22 months after the initial stress, with colonies reduced by 47.2% (±15.9 *SE*) from their 2005 extent. Unlike *O. franksi*, the unbleached group (*n* = 6) also had extensive tissue loss, also reaching the lowest point in month 22, with a 51.9% (*SE* ±44.8) reduction in live area. *S. michelini* had the highest colony mortality following the bleaching, with 16 of the 68 colonies (23.5% of the total sample) dead in the first 9 months. All but one of these colonies were bleached in 2005, with an average bleaching area for this group of 56.7% (±25.1 *SE*), not significantly different than the mean for the bleached group itself (*t *=* *0.7195, *df* = 122, *p *=* *.4732). For comparison, only one additional colony died over the next 49 months.

*Siderastrea siderea* showed the least amount of tissue loss per colony, with the bleached colonies (*n* = 25) losing an average of 11.2% (±3.9 *SE*) of live tissue area per colony in the first nine months following the bleaching event (Table 2). However, unlike the other two species, live tissue area continued a slow but even decline across the remaining period, and did not return to positive growth. The unbleached group (*n* = 33) did not have significant initial tissue loss in response to the thermal stress event, losing <1% in the first 6 months, but this group subsequently showed a steady loss of tissue area over the remaining time, similar to the bleached coral group. *S. siderea* had the lowest total colony mortality, with no colonies dying in the 9 months following the bleaching, and only one colony having documented total mortality over the first 58 months of the study. At that time, 52 of the original 58 live colonies remained in the record, with five colonies lost to the record, presumably through tag shedding, failure to relocate, or other unknown causes. These corals were eliminated from the record and therefore do not affect subsequent year‐on‐year changes.

### 3.4 Recovery of tissue pigmentation following bleaching

Bleached *O. franksi* colonies had a reduction in mean bleached area to <1% of remaining live area by 6 months following the 2005 bleaching event and did not exceed this threshold in any subsequent observations. Partially bleached area returned to normal pigmentation more slowly, with colonies in the bleached group still showing 12.4% (±1.1 *SE*) of their area partially bleached after 9 months. In the unbleached control group, neither mean bleaching nor partial bleaching exceeded 2.1% (±0.5 *SE*) in any of the observations at any point in the time series (Table 3).

**Table 3:** Live coral cover extent and changes in total coral cover area

Species	Status	Total live area sampled in November 2005, cm^2^	Live area 9 months postbleaching, cm^2^	% Live area 9 months postbleaching/2005 area	Live area 58 months postbleaching, cm^2^	% Live area 58 months postbleaching/2005 area	Live area 89 months postbleaching, cm^2^	% Live area 89 months postbleaching/2005 area
*Orbicella franksi*	All	27,699.51	19,975.11	72.11	19,849.09	71.66	15,946.01	57.57
*O. franksi*	Unbleached	5,802.62	6,017.88	103.71	5,743.75	98.99	4,593.62	79.16
*O. franksi*	Bleached	21,896.89	13,957.23	63.74	14,105.34	64.42	11,352.39	51.84
*Stephanocoenia michelini*	All	16,318.98	10,198.80	62.50	96,15.47	58.92	5,053.71	30.97
*S. michelini*	Unbleached	1,244.40	1,128.38	90.68	901.83	72.47	457.44	36.76
*S. michelini*	Bleached	15,074.58	9,070.42	60.17	8,713.64	57.80	4,596.27	30.49
*Siderastrea siderea*	All	22,271.94	18,959.23	85.13	16,619.49	74.62	15,809.24	70.98
*S. siderea*	Unbleached	14,563.45	12,237.94	84.03	11,697.08	80.32	11,097.40	76.20
*S. siderea*	Bleached	7,708.49	6,721.29	87.19	4,922.41	63.86	4,711.84	61.13

Includes the total study sample, from both response (between 0 and 9 months after bleaching) and recovery (between 22 and 58 months after bleaching) periods. Totals shown for each species include both the unbleached and bleached colony groups for those species.

For bleached *S. michelini* colonies, bleached tissue extent declined from 39.5% (±5.0 *SE*) to 1.1% (±0.1 *SE*) by 6 months following the initial bleaching. A notable extent of partially bleached area was recorded for this species throughout the time series, dropping to a low of 5.1% 34 months after the bleaching event, but rising again in 2011 to 15.9% of live area. Colonies in the unbleached group notably showed increasing areas of partially bleached tissue in observations subsequent to the initial event, roughly tripling in area by the month 6 of the study to 15.5% (±2. *SE*), and declining thereafter. Bleached area rose to a post‐2005 high of 3.1% (±0.4 *SE*) 11 months after the second thermal event, with the unbleached group also showing a increase in bleached area in 2011 (Table 3).

The mean bleached tissue extent of bleached *S. siderea* colonies was less than the other species and also declined more slowly, from nearly 13.1% (±2.6 *SE*) in the initial observation to 3.0% (±0.6 *SE*) by 9 months. Bleached area in subsequent observations remained low (~1%) and stayed at that level through the second thermal event. Colonies in the unbleached group maintained low areas of both bleached and partially bleached area throughout the time series (Table 3).

### 3.5 Colony growth following initial bleaching/stress event

The period from month 22 to month 56 was unaffected by major water temperature anomalies, and it is assumed that there were little or no direct physiological impacts on the corals from thermal stress during this time (Stephenson et al., 2014). This lack of physiological impact is inferred from the temperature record and was not measured *in situ*. This time period is taken to be indicative of normal environmental conditions and thus potentially represents colony tissue expansion and growth unaffected by thermal stress and is refereed to below as the recovery period.

Annualized mean colony tissue expansion for the unbleached *O. franksi* group in the recovery period was 1.9%, an increase from the essentially flat annualized tissue growth of −0.53% in the first 9 months following the thermal stress event. Variance in this group increased over time, as would be expected with a time series, as growth trajectories for individuals vary, but was not large in the initial years, indicating consistent initial growth and mortality response within the unbleached group, as expected. The bleached group of *O. franksi* also showed a return to positive live tissue area expansion in the recovery period, increasing in mean live area by a mean annual rate of 2.23% during the period, after having decreased at an annualized rate of −40.3% for the first 9 months.

Bleached *S. michelini* colonies ceased to decline after initially losing large amounts of tissue (−56.6% annualized decline) and experienced an essentially flat tissue growth rate (0.05%) for the recovery period. In contrast to the flat positive tissue expansion response of the unbleached *O. franksi* colonies, the unbleached *S. michelini* group declined in both the first 9 months and also in the recovery period, although at a reduced rate in the later period. However, this group is not large enough to be considered representative (*n* = 6), and variance for this set of samples was high for all years.

For *S. siderea,* both bleached and unbleached groups of colonies slowly declined in live area across response and recovery time periods. After initially larger tissue loss, the bleached group reduced in area at a similar gradual annualized rate (−1.66%; *n* = 21) to the unbleached group (−3.33%; *n* = 31) during the recovery time. This slow but steady decline characterized the response of this species across the whole study period; with neither group showing positive growth maintained over any three observation periods for any point in the time series.

### 3.6 Response to the second thermal stress event in 2010

The two observation points following the 2010 elevated water temperature event cover 29 months after the event, with measurements taken in September 2011 (11 months after the maximum thermal stress point) and March 2013. The 2010 photographic observation and sample collection does not represent the corals in a stressed condition, as the field observations preceded the warmest period of the summer by about 1 month, and thus missed the period of greatest bleaching extent that year.

**Figure 5:**
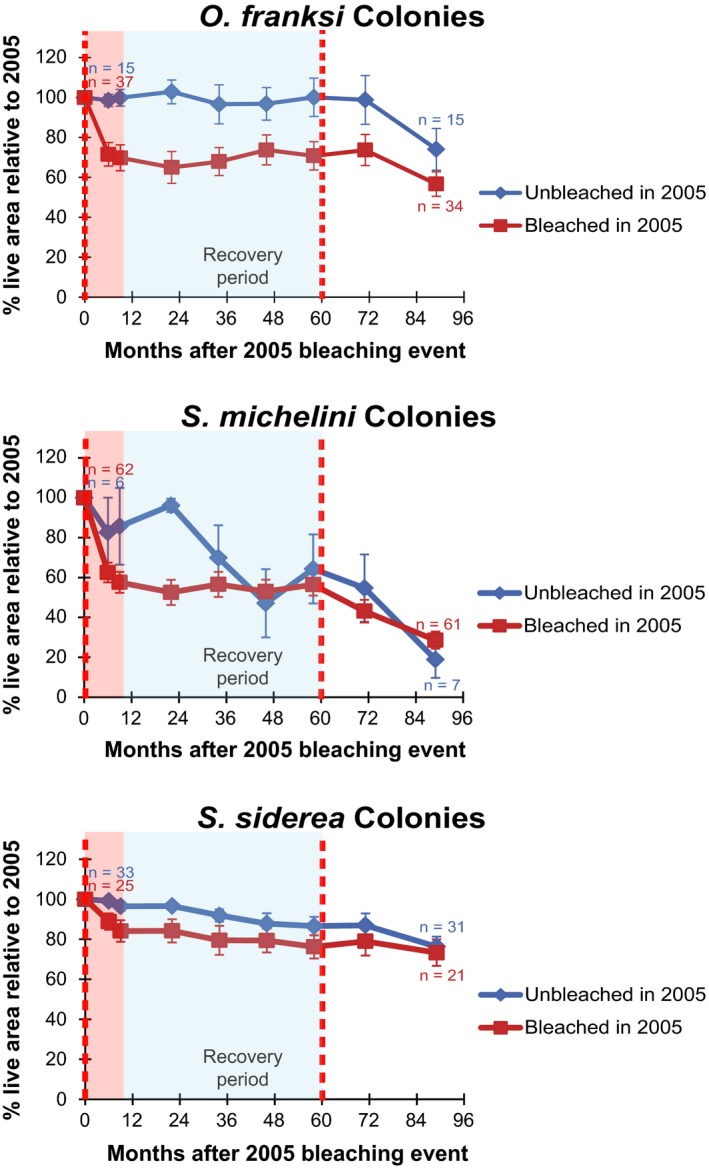
Percent change in live area of colony tissue relative to 2005 (the initial bleaching event) for bleached and unbleached colony groups. Colonies that bleached in 2005 are expressed as the dark red line and unbleached colonies as the dark blue. The red dashed lines correspond to the mass bleaching events in 2005 (month 0) and 2010 (Month 60). The bleaching response period is highlighted in light red, and the recovery period highlighted in light blue. Error bars represent standard error. Bleached colonies of *O. franksi* and *S. michelini* lost more live area than unbleached colonies following the first bleaching event in 2005. After the second bleaching event in 2010, however, the colonies unbleached in 2005 lost more tissue than those previously bleached in 2005

Both the initially bleached and unbleached groups of *O. franksi* colonies exhibited a decline of live tissue in both 2011 and 2013, compared to 2010 areas (prior to the bleaching). The annualized live tissue area change rate for the period from month 58–89 for previously bleached group was −9.1% and for the previously unbleached was −12.7%. Both the initially bleached and unbleached groups of *S. michelini* colonies also exhibited loss of live tissue in both 2011 and 2013, compared to 2010 areas, at greater rates than *O. franksi*. The annualized live tissue area change rate for the period from month 58–89 for the previously bleached group was −20.6% and for the previously unbleached was −26.4%. *S. siderea* colonies showed little response to the second thermal stress event, with both groups continuing a slow decline at a similar rate to the earlier periods. The annualized live tissue area change rate for the period from month 58–89 for the previously bleached group was −3.4% and for the previously unbleached group was −3.7% (Figure 5).

## 4 Discussion

### 4.1 Response to thermal stress varies by species

The extent of bleaching in the initially bleached group for each of the three species varied widely. Recognizing our aim to quantify species‐specific bleaching recovery dynamics (and not overall taxon‐specific bleaching susceptibility—i.e., the extent of bleaching within a representative population), our findings indicate that *S. michelini* and *O. franksi* were highly susceptible to severe bleaching, while *S. siderea*. appeared highly bleaching resistant. This notable difference in visible response, along with the inherent subjectivity of field identification of “bleached” individuals, brings into question how the widely used designation of “bleached” should be defined for different species and raises the need for more sensitive field methods for the diagnosis of onset of coral stress, such as *in situ* fluorescence (Treibitz et al., 2015), measurements of cellular apoptosis (Ainsworth, Hoegh‐Guldberg, Heron, Skirving, & Leggat, 2008), or concentrations of heat shock proteins (HSP) (Rosic, Pernice, Dove, Dunn, & Hoegh‐Guldberg, 2011). Given that *S. siderea* continued to show a decline in live tissue extent across the remainder of the study period, the lack of visible bleaching symptoms during the initial stress event may not indicate a resistance to thermal stress‐related impacts, but simply a lack of immediate visual response to thermal stress. Similar taxon‐specific variable response by corals to thermal stress has been previously demonstrated (Anthony, Kline, Diaz‐Pulido, Dove, & Hoegh‐Guldberg, 2008; Marshall & Baird, 2000). Many factors other than coral species can confer significant within‐ and between‐species variation in response to thermal stress (Brandt, 2009), including symbiont phylotype (Van Oppen, Baker, Coffroth, & Willis, 2009), size‐structured or age‐related differences in a population (Brown et al., 2014), differences in thermal stress with depth (Neal et al., 2010), local stressors such as sedimentation (Carilli et al., 2009), CO_2_ exposure (Anthony et al., 2008), or historical exposure to water temperature variation (Carilli, Donner, & Hartmann, 2012). Further species‐specific effects may differ between reef sites, as individual colony history, environmental exposure, and benthic and community structure vary.

### 4.2 Area of partial mortality over time following bleaching was closely related to total initial bleached area

Partial mortality in the 9 months following the 2005 bleaching disturbance also varied widely by species and was closely correlated with the initial amount of bleaching in that species. This suggests that areal bleaching extent in an affected coral colony is a strong predictive indicator for magnitude of subsequent eventual tissue loss. Tissue loss was greatest for *S. michelini,* followed by *O. franksi,* and was nearly negligible for *S. siderea* colonies. Maximum partial mortality was not fully reached in the first 6 months of the study, indicating the importance of following recovery of bleached corals for longer than the time span needed for symbiont repopulation, and suggests a persistent reduction in colony function may be incurred through thermal stress. This delayed response was first reported by Glynn in 1990 (Glynn & D'croz, 1990), noting that following the 1982–1983 bleaching event in the eastern Pacific several massive‐type corals (*Porites panamensis*) regained normal visible appearance and coloration within 2–3 months after bleaching, but then experienced total colony mortality between 7 and 10 months. Our findings indicate the this postbleaching mortality window could be as long as a year for *S. micheleni* and *O. franksi,* which occupy similar niches in the Caribbean to *Porites* spp. in the Pacific, and that the time frame for reduced growth following thermal exposure could be up to 3 years.

### 4.3 Recovery from bleaching possible in thermally stable conditions

Growth rates during the 4‐year recovery period are assumed to represent growth rates for these species under nonstressful temperature conditions. This assumption appears to be potentially valid for *S. michelini* and *O. franksi,* but not for *S. siderea,* which demonstrated a slow but regular decline across the study period. Furthermore, the relatively small growth rates we report here, and the large variance in growth across colonies, point out the difficulty in determining true growth rates for massive‐type corals, which must be measured over many years, i.e., longer than the 7 years of this study. Given inherent methodological variance in measuring planar area from photographs (Neal et al., 2013) and the absence in the record of some colonies in some years due to tag shedding or missed photographs, the narrowly positive rates we report may not be maximum individual growth rates for these corals, but could alternatively indicate net maintenance of tissue (i.e., a flat growth rate) which may persist for many years. In either case, these even or positive rates for the recovery period do provide a good comparison to the marked losses of the initial and response periods.

The consistent tissue loss for *S. siderea* remains unexplained and may be related to other factors than temperature. It may be due to senescence from age, competitive interactions or predation, or effects from unmeasured environmental factors such as ocean acidification, sedimentation, pollution, disease, or corallivory preferentially affecting this species. However, senescence or targeted predation appears unlikely given the relatively small sizes of our colonies (39–1133 cm^2^; mean = 383.9 cm^2^ (±37.5 *SE*)), compared to recorded larger sizes for this species (Lewis, 1997). Furthermore, the regularity of tissue loss across our samples and the rarity of total mortality across the 89 months (only six colonies dead or lost, from *n* = 58 in 2005) indicate a possible chronic force at work. The steady decline of only *S. siderea* in this location, for all years, and similar in both bleached and unbleached groups, suggests that it may be unrelated to the temperature anomalies or bleaching event, or may be a synergistic effect from bleaching stress combined with other factors. In particular, ocean acidification (OA) may manifest with such slow but persistent impacts (Shaw, Phinn, Tilbrook, & Steven, 2015). Furthermore, the effect of altered growth or persistence on specific members of the reef community may affect overall net reef deposition (Shaw, Hamylton, & Phinn, 2011); one indication of this is that areas of the northern Florida Reef Tract may already be in a condition of negative net community calcification (Muehllehner et al.*,* 2016). The species‐specific differences in postdisturbance recovery documented here increase capacity for improved modeling of growth and calcification dynamics for reef systems.

*Stephanocoenia michelini* and *Orbicella franksi* both demonstrated positive expansion of live tissue area during the recovery period, for all four test groups (i.e., for both species, in both previously bleached and unbleached groups). The sample size of unbleached *S. michelini* is too small to be representative, as seen in the high variance in this record, but the other three groups showed steady growth in live area. Interestingly, the live tissue area expansion rates for both previously bleached groups exceeded those of the previously unbleached colonies, possibly indicating a positive acclimation related to bleaching exposure. This resumption of positive live tissue aggregation is clearly important for ecosystem recovery from disturbance, but even though there was expansion in all groups, only one group (unbleached *O. franksi*) grew beyond the aggregate live tissue area of 2005. The other groups, while showing positive annual growth, were still, after 8 years, recovering from tissue losses directly resulting from the 2005 event.

The expansion in live coral tissue area shown during the recovery period indicates that massive‐type coral colonies in the Caribbean can resume positive growth following severe thermal disturbance and bleaching, and demonstrably can continue this growth if thermal stress conditions are not present. However, even with the most positive tissue expansion rates demonstrated (for the bleached sets of *S. michelini* and *O. franksi*), the extrapolated time periods for complete recovery of the area of live tissue lost to the 2005 event are on the order of many decades (32–128 years), assuming no other disturbances occur in this time. These lengthy time periods are an indicator of the need to account for hysteresis and nonlinear dynamics in estimating recovery dynamics for postdisturbance coral ecosystems. With a second stress event experienced in this location within 5 years, during which the corals in this study had not recovered from initial 2005 tissue losses, ensuring decadal‐scale recovery times for coral reefs from acute thermal stress appears unlikely. Elevated water temperatures are currently affecting corals in this area at the time of this writing (late 2015–early 2016), further demonstrating contemporary reduced time frames between thermal stress events.

Both the time periods taken to return to net positive growth and the theoretical time frames needed to recover to initial sample sizes are significant. Extrapolating between observation periods for the *O. franksi* bleached group (which showed the highest net tissue aggregation rate for any group in this study) indicates that net positive growth would have been reached ~33.6 months following the disturbance (second‐order polynomial regression, *r* ^2^=0.85), and that recovery to a total live area equal to the start of the study (assuming maximum mean growth rate continued undisturbed) would have taken ~32.6 years. For *S. michelini,* a return to net positive growth for the bleached group was reached ~36.5 months (second‐order polynomial regression, *r* ^2^ = 0.51), and with the maximum growth rate experienced in the recovery period would have required 128.8 years to return to a total live area prior to 2005. For *S. siderea,* no estimated time to return to positive growth following the initial stress event, as the group continued to decline. Using mean tissue area loss rates for both bleached and unbleached groups, live area for this species will be reduced to <50% of original extent in 22 years and would be <10% of the original area within 91 years. These extrapolations are intended to be illustrative only as heuristic demonstrations, as they assume steady environmental conditions, likely not a valid assumption, but they do demonstrate the notable hysteresis in recovery trajectories from acute coral bleaching events, and the need for long recovery periods between these disturbances, a need that is not being met under current conditions.

### 4.4 Reduced thermotolerance seen in second stress event for previously unbleached corals

The reductions in colony area seen in 2011 and 2013 appear to be an effect of the second thermal stress event in 2010. Like the 2005 event, there was surprisingly little total colony mortality, but there was marked loss of tissue and continued fragmentation for nearly all colonies monitored. This reduction was seen in both previously bleached and unbleached groups for all three species, although we focus the discussion on *S. michelini* and *O. franksi,* as the decline for *S. siderea* was a continuation of the trend seen before the second stress event, which was seemingly little affected by either the first or second thermal stress event. For both *S. michelini* and *O. franksi,* the previously unbleached colonies declined more than the previously bleached groups, possibly indicating that prior thermal stress exposure alone does not confer the same level of acclimative advantage as prior visible bleaching. Silverstein et al. (2016) demonstrated this concept in the laboratory over a time frame of approximately 1 year, and it is compelling to find this effect also seemingly demonstrated 5 years after the initial exposure. This acclimative effect on previously bleached corals has been described as a possible “nugget of hope” for coral reefs in times of climate change, and our results do provide possible *in situ* support for the concept that previous bleaching can confer a possible benefit to the short‐term survival of individual colonies of some scleractinian species, and thus to the long‐term survival of coral reefs as growing, structural ecosystems (Berkelmans & van Oppen, 2006). However, this acclimation must be viewed within a temporal context that allows this benefit to be fully expressed, which may not be the case for our study period, or for the projected future.

### 4.5 Previously bleached and unbleached corals had similar cumulative mortality when exposed to repeat thermal stress

Another surprising finding supporting the idea that acclimative benefits were limited in their overall impact on coral survival and growth was that the total live area for each of the conspecific groups of previously bleached and previously unbleached colonies were not significantly different after the 89 months of the study. This was true for all three species, despite notable differences in growth response and total area between some of the bleached and unbleached groups throughout this study. This similar tissue decline in tissue area seen by the endpoint of the study is likely attributable in two of the species (*S. michelini* and *O. franksi*) to the effects of the second thermal stress event on the previously unbleached groups. This is most clearly seen in *O. franksi,* where the previously unbleached group had no tissue loss following the initial thermal disturbance and demonstrated steady growth for a number of years immediately following this event, but the tissue area gains accumulated during this period were subsequently completely lost following the 2010 stress event, and this group was not larger than the previously bleached group, which was still making up area lost to direct mortality in 2005. In other words, while the survival of colonies previously exposed to severe high‐stress thermal exposure without visible bleaching does incur an immediate clear benefit, as there is less immediate partial colony mortality, but if there is further stress this may be an ephemeral advantage, as they appear at greater risk for larger losses in subsequent bleaching events. With thermal stress conditions occurring in this ecosystem in 1998 (Glynn, Maté, Baker, & Calderón, 2001), 2005 (Eakin et al., 2010), 2010 (Levitan, Boudreau, Jara, & Knowlton, 2014), and potentially (at the time of writing) in late 2015, the time between bleaching events could likely be less in the future than the needed recovery periods indicated by these results, meaning that cumulative mortality from multiple events could possibly overwhelm any benefit conferred by either “escape” from bleaching or from individual acclimation from prior bleaching.

The concept that bleaching is exceeding the inherent necessary recovery period for these corals is supported by this study as all three species (in aggregate) ended the time series having experienced net loss of live tissue, regardless of prior bleaching history. This outcome did vary by species, but the living tissue decline for all of these three critical ecosystem‐structuring species when faced with repetitive thermal stress suggests that Caribbean reefs may face massive challenges in a warmer future. If thermal stress and bleaching events increase in frequency and severity, and there is no acclimative increase in colony resilience, then the future persistence of massive‐type corals in the western Caribbean appears possibly uncertain. While the rates of chronic decline shown in this study are annually still low, they indicate the ecologically chatastrophic possibility of reduction in live coral cover in the Caribbean to functionally collapsed levels within time frames of a few decades. Coral holobiont communities (specifically including associated symbiont communities) do appear to have rapidly formed specific associations conferring greater thermal tolerance in limited areas subjected to rapid and extreme environmental change, such as in the Persian/Arabian Gulf in the Holocene (Hume et al., 2016), but in that case the time frame was ~1–6 thousand years. While this example is rapid by evolutionary standards, it is not nearly as rapid as current environmental change. Given the possible reduction in both diversity and community extent suggested in this work as the result of repeated thermal stress, existing coral host and symbiont biodiversity and reproductive success may simply not be maintained in these systems over a sufficiently long time frame to allow for stress‐tolerant associations to evolve and persist.

## Conflict of Interest

None declared.
